# Performance of glomerular filtration rate estimation equations in Congolese healthy adults: The inopportunity of the ethnic correction

**DOI:** 10.1371/journal.pone.0193384

**Published:** 2018-03-02

**Authors:** Justine B. Bukabau, Ernest K. Sumaili, Etienne Cavalier, Hans Pottel, Bejos Kifakiou, Aliocha Nkodila, Jean Robert R. Makulo, Vieux M. Mokoli, Chantal V. Zinga, Augustin L. Longo, Yannick M. Engole, Yannick M. Nlandu, François B. Lepira, Nazaire M. Nseka, Jean Marie Krzesinski, Pierre Delanaye

**Affiliations:** 1 Renal Unit, Department of Internal medicine, Kinshasa University Hospital, University of Kinshasa, Kinshasa, Democratic Republic of the Congo; 2 Division of Clinical Chemistry, CHU Sart Tilman (ULg CHU), University of Liège, Liège, Belgium; 3 Division of Public Health and Primary Care, KU Leuven Campus Kulak Kortrijk, Kortrijk, Belgium; 4 Division of Nephrology-Dialysis-Transplantation, CHU Sart Tilman (ULg CHU), University of Liège, Liège, Belgium; University of Colorado Denver School of Medicine, UNITED STATES

## Abstract

**Context and objective:**

In the estimation of glomerular filtration rate (GFR), ethnicity is an important determinant. However, all existing equations have been built solely from Caucasian and Afro-American populations and they are potentially inaccurate for estimating GFR in African populations. We therefore evaluated the performance of different estimated GFR (eGFR) equations in predicting measured GFR (mGFR).

**Methods:**

In a cross-sectional study, 93 healthy adults were randomly selected in the general population of Kinshasa, Democratic Republic of the Congo, between June 2015 and April 2016. We compared mGFR by plasma clearance of iohexol with eGFR obtained with the Modified Diet in Renal Disease (MDRD) equation with and without ethnic factor, the Chronic Kidney Disease Epidemiology (CKD-EPI) serum creatinine (SCr)-based equation, with and without ethnic factor, the cystatin C-based CKD-EPI equation (CKD-EPI SCys) and with the combined equation (CKD-EPI SCrCys) with and without ethnic factor. The performance of the equations was studied by calculating bias, precision and accuracy within 30% (P30) of mGFR.

**Results:**

There were 48 women and 45 men. Their mean age was 45.0±15.7 years and the average body surface area was 1.68±0.16m^2^. Mean mGFR was 92.0±17.2 mL/min/1.73m^2^ (range of 57 to 141 mL/min/1.73m^2^). Mean eGFRs with the different equations were 105.5±30.1 and 87.2±24.8 mL/min/1.73m^2^ for MDRD with and without ethnic factor, respectively; 108.8±24.1 and 94.3x20.9 mL/min/1.73m^2^ for CKD-EPI SCr with and without ethnic factor, respectively, 93.5±18.6 mL/min/1.73m^2^ for CKD-EPI SCys; 93.5±18.0 and 101±19.6 mL/min/ 1.73m^2^ for CKD-EPI SCrCys with and without ethnic factor, respectively. All equations slightly overestimated mGFR except MDRD without ethnic factor which underestimated by -3.8±23.0 mL/min /1.73m^2^. Both CKD-EPI SCr and MDRD with ethnic factors highly overestimated mGFR with a bias of 17.9±19.2 and 14.5±27.1 mL/min/1.73m^2^, respectively. There was a trend for better P30 for MDRD and CKD-EPI SCr without than with the ethnic factor [86.0% versus 79.6% for MDRD (p = 0.21) and 81.7% versus 73.1% for the CKD-EPI SCr equations (p = 0.057)]. CKD-EPI SCrCys and CKD-EPI SCys were more effective than creatinine-based equations.

**Conclusion:**

In the Congolese healthy population, MDRD and CKD-EPI equations without ethnic factors had better performance than the same equations with ethnic factor. The equations using Cys C (alone or combined with SCr) performed better than the creatinine-based equations.

## Introduction

Nowadays, chronic kidney disease (CKD) has been recognized as a major public health burden [1, 2]. In sub-Saharan Africa (SSA), CKD increased healthcare costs and is emerging as one of the major health threats contributing every year to millions of premature deaths. It affects young adults in their productive years, particularly in high risk groups of people with hypertension, diabetes, obesity, sickle cell disease or HIV infection [3–8]. Early detection and timely intervention of CKD can prevent or delay these adverse outcomes [9, 10]. Thus, the increased prevalence of CKD emphasizes the need to establish appropriate and well validated methods to assess renal function. Proteinuria and glomerular filtration rate (GFR) are generally considered as the best indicators of kidney function [11]. The ideal method to evaluate GFR is by measuring clearance of an exogenous marker, such as inulin or iohexol, but this is costly and time-consuming [12, 13]. Because measuring GFR is relatively cumbersome, more simple biologic variables have been proposed based on serum creatinine (SCr) and demographic factors such as age, body size and sex [14, 15]. Available guidelines recommend the use of creatinine-based equations, such as modification of diet in renal disease (MDRD) [15, 16] and CKD epidemiology collaboration (CKD-EPI), to estimate GFR [17]. To recognize CKD early and to properly manage kidney disease, the 2012 Kidney Disease Improving Global Outcomes (KDIGO) recommend using SCr and/or cystatin C-based equations to estimate GFR [18]. Recent studies have shown that estimated GFR using serum cystatin C (eGFR SCys) is not more precise than estimated GFR using SCr (eGFR SCr), whereas estimated GFR based on both markers (eGFRSCrCys) is more precise than eGFR based on either marker alone [19–21]. The limitation is linked to the fact that SCr concentration is dependent not only on GFR but also on muscular mass. Given the strong link to muscular mass, SCr concentration and creatinine excretion will vary with socioeconomic status, physical activity, nutrition, gender, age and ethnicity, independently of any GFR changes [22, 23]. For this reason, creatinine-based equations have been developed, including these last three variables to estimate GFR [14, 15, 21]. SCr concentration will also differ between ethnicities for the same GFR level because it is suspected that African people proportionally have a larger muscular mass than Caucasian or Asian populations [24–26]. Furthermore, some authors suggested that tubular secretion of creatinine could be different according to ethnicity [23, 27], but this is still controversial [28]. Hence, MDRD and CKD-EPI-based equations proposed to use a correction factor for black Americans. Non critical application of these equations in SSA individuals appears questionable and highlights the need for their validation. Several studies have questioned the application of this ethnic factor to black Africans, particularly South-Africans, Ghanaians and Ivoirians [26, 28–30]. However, to date, there is no data showing the comparison of equations using creatinine alone or combined with cystatin C (with or without ethnic factor) with mGFR in the Central Africa population. Therefore, we assessed the performance of MDRD and different CKD-EPI equations in Congolese adults.

## Methods

### Study design and population

This current cross-sectional study is the fourth part of a larger ongoing epidemiological survey on CKD and associated risk factors termed ‘‘Prévalence, détection précoce et prévention des maladies rénales chroniques et facteurs de risque associés (PDMRA) en République Démocratique du Congo”, carried out between June 2015 and April 2016, in the general population of Kinshasa, capital of the Democratic Republic of the Congo (DRC).

All participants were visited twice at home by trained research personnel, who recorded information on demographics, diet, smoking, alcohol consumption, and use of indigenous herbal remedies. Data about family relatives of first degree and medical history for kidney disease, hypertension, diabetes and current treatment were also recorded. Body weight, height and abdominal circumference were measured.

The healthy persons were recruited from the general population as mentioned above. Before inclusion in the study for measurement of GFR by plasma clearance of iohexol, these subjects were screened for the absence of acute and chronic pathologies including hypertension, diabetes mellitus, obesity, cardiovascular diseases, renal dysfunction (eGFR CKD-EPI SCr < 60 or > 130 mL/min/1.73m^2^), and urinary abnormalities (proteinuria, hematuria or leucocyturia) by urine dipstick (“Combur10 Test”, Roche Diagnostics, Mannheim, Germany) on the morning urine. Participants were asked to avoid medications that affect GFR (e.g. anti-inflammatory agents, diuretics, renin-angiotensin blocking agents) and those that can interfere with creatinine secretion (e.g. cimetidine or trimetroprim). Briefly, medication was not allowed except for contraceptives in the female participants.

The study protocol was approved by the Ethics Committee of the Public Health School of the University of Kinshasa (N°ESP/CE/029/2015) and by the Institutional Review Boards at each site. Written informed consent was obtained from all participants, and all participants with abnormal findings received counseling, and educational pamphlets.

### GFR measurement and estimation

GFR was measured using iohexol plasma clearance (Omnipaque, 240 mg I/mL, GE Healthcare, Belgium) as the method of reference once in every patient [12, 13, 31]. After verification of the participant’s identity, a rest of 5 minutes was observed. The syringe with iohexol was weighed before and after the injection to an accuracy of 0.01 g. In the morning, a catheter was placed in a large vein of the forearm, and 5 mL of blood was taken at time 0 for the determination of both SCr and cystatin C. By the same vein, 5 ml of iohexol was injected before removing the catheter and the empty syringe was weighed. Then 4 blood samples were drawn from a different intravenous access (usually from the contralateral arm) at 120, 180, 240 and 300 minutes after the injection of iohexol [29]. The blood sample was allowed to stand for 30–60 minutes before being centrifuged. After centrifugation, samples were stored at -80°C. Then, samples were shipped for iohexol measurement at the University laboratory of Liège, Belgium and then measured by liquid chromatography-tandem mass spectrometry (LC-MS/MS), as previously described [32]. To assure the quality of iohexol measurements, the laboratory is accredited for the ISO 15189 Standard and also participates in the interlaboratory quality test for iohexol performed by Equalis AB, Uppsala, Sweden. The measured GFR (mGFR) was then calculated using the slope-intercept method, corrected with the Brochner-Mortensen equation [12]. In our hands, the intra-individual coefficient of variation for iohexol plasma clearance was 4.5% [12]. The results were normalized by body surface area (BSA) using the Gehan and Georges [33] formula as follows: BSA = 0.0235 x weight ^0.51456^ x height ^0.42246^.

GFR (mL/min/1.73m^2^) was also estimated by using different equations as described in Table 1.

**Table 1:** Creatinine and cystatin C-based equations for glomerular filtration rate estimation.

MDRD		175 x Scr^-1.154^ x age^-0.203^ x [0.742 if female] x [1.212 if black]
CKD-EPI SCr		
Female	SCr ≤ 0.7 mg/dL	144 x (Scr/0.7)^-0.329^ x 0.993^age^ x [1.159 if black]
	SCr > 0.7 mg/dL	144 x (Scr/0.7)^-1.209^ x 0.993^age^ x [1.159 if black]
Male	SCr ≤ 0.9 mg/dL	141x (Scr/0.9)^-0.411^ x 0.993^age^ x [1.159 if black]
	SCr > 0.9 mg/dL	141x (Scr/0.9)^-1.209^ x 0.993^age^ x [1.159 if black]
CKD-EPI SCys	SCys ≤ 0.8 mg/L	133 x (Scyst/0,8)^-0.499^ x 0.996^age^ [x0.932 if female]
	SCys >0.8 mg/L	133 x (Scys/0,8)^-1.328^ x 0.996^age^ [x0.932 if female]
CKD-EPI SCrCys		
Female	SCr ≤ 0.7 mg/dL and SCys ≤ 0.8 mg/dL	130 x (Scr/0.7)^-0.248^ x (Scyst/0.8)^-0.375^ x 0.995^age^ x [1.08 if black]
	SCr ≤ 0.7 mg/dL and SCys > 0.8 mg/dL	130 x (Scr/0.7)^-0.248^ x (Scyst/0.8)^-0.711^ x 0.995^age^ x [1.08 if black]
	SCr>0.7 mg/dL and SCys ≤ 0.8 mg/dL	130 x (Scr/0.7)^-0.601^ x (Scyst/0.8)^-0.375^ x 0.995^age^ x [1.08 if black]
	SCr >0.7 mg/dL and SCys ≥ 0.8 mg/dL	130 x (Scr/0.7)^-0.601^ x (Scyst/0.8)^-0.711^ x 0.995^age^ x [1.08 if black]
Male	SCr ≤0.9 mg/dL and SCys ≤ 0.8 mg/dL	135 x (Scr/0.9)^-0.207^ x (Scyst/0.8)^-0.375^ x 0.995^age^ x [1.08 if black]
	SCr≤0.9 mg/dL and SCys > 0.8 mg/dL	135 x (Scr/0.9)^-0.207^ x (Scyst/0.8)^-0.711^ x 0.995^age^ x [1.08 if black]
	SCr >0.9 mg/dL and SCys ≤ 0.8 mg/dL	135 x (Scr/0.9)^-0.601^ x (Scyst/0.8)^-0.375^ x 0.995^age^ x [1.08 if black]
	SCr >0.9 mg/dL and SCys > 0.8 mg/dL	135 x (Scr/0.7)^-0.601^ x (Scyst/0.8)^-0.711^ x 0.995^age^ x [1.08 if black]

KD-EPI SCr: Chronic Kidney Disease-Epidemiology Collaboration equation based on serum creatinine only; CKD-EPI SCys: CKD-EPI equation based on cystatin C only; CKD-EPI SCrCys: CKD-EPI combining creatinine and cystatin C; MDRD: Modification of Diet in Renal Disease study equation. SCr: serum creatinine (in mg/dL); SCys: Serum Cystatin C (in mg/L).

SCr levels were determined in the same laboratory by using an enzymatic method with an IDMS-traceable calibrator using the Roche Cobas (Roche Diagnostics, Mannheim, Germany). Serum Cystatin C levels were measured by the latex immunoturbidimetric method using Roche Cobas (Roche Diagnostics, Mannheim, Germany Mannheim, Germany) and standardized to ERM-DA 471/IFCC [34].

### Statistical analysis

All analyses and calculations were performed using SAS 9.4 (SAS Institute Inc., Cary, NC, USA). Data were expressed as mean ± standard deviation. The comparison of the mean of two or more groups was made using Student-t-test and ANOVA, respectively. We assessed the performance of the MDRD and CKD-EPI eGFRs with and without ethnic factors using the following statistical tools. Lin’s Concordance Correlation Coefficient (CCC) evaluated the degree to which pairs of observations fall on the 45° line through the origin. It’s a measure of both correlation and agreement. Spearman correlation coefficients were added in the S1 File. Then, bias (the difference between eGFR- mGFR, systematic error) and relative bias were calculated. Precision was evaluated by the standard deviation of the bias (random error) and root-mean-square error (RMSE) (the square root of the mean squared differences eGFR-mGFR). Moreover, we considered the accuracy within 30% and 10%, i.e. the percentage of estimates falling within ±30% (P30) or ±10% (P10) of measured GFR [35, 36]. The difference in P30 accuracy between eGFRs was tested with the exact McNemar test. In S2 and S3 Files, sub-analyses according to gender and GFR tertiles were done.

eGFR determined by each equation was finally compared with mGFR using the Bland-Altman analysis. The threshold of statistical significance was set at α = 0.05.

## Results

### General characteristics of study population

Table 2 shows general characteristics of the participants and according to sex.

**Table 2:** General characteristics of study population by sex.

Variables	Overall n = 93	Male n = 45	Female n = 48	P value
Age (years)	45.0±15.7	46.1±16.9	44.0±14.5	0.531
Height. (cm)	163.8±8.9	169.0±8.1	158.9±6.5	**<0.001**
Weight. (kg)	63.1±10.5	64.5±9.6	61.7±11.2	0.207
BMI (kg/m^2^)	23.5±3.4	22.6±3.1	24.3±3.4	**0.011**
BSA (m^2^)	1.68±0.16	1.74±0.15	1.63±0.16	**0.0013**
Serum Creatinine (mg/dL)	0.87±0.18	0.95±0.19	0.80±0.15	**<0.001**
Cystatin C (mg/L)	0.90±0.14	0.95±0.13	0.85±0.14	**0.001**
mGFR (mL/min/1.73m^2^)	92.0±17.2	95.5±15.8	88.8±17.9	0.0603
MDRD (mL/min/1.73m^2^)	105.6±30.1	112.0±35.7	99.5±22.4	**0.044**
MDRD nef (mL/min/1.73m^2^)	87.2±24.8	92.6±29.4	82.1±18.5	**0.044**
CKD-EPI SCr nef (mL/min/1.73m^2^)	94.3±20.9	96.6±21.1	92.1±20.6	0.309
CKD-EPI SCr (mL/min/1.73m^2^)	109.3±24.2	111.9±24.5	106.8±23.9	0.309
CKD-EPI SCys (mL/min/1.73m^2^)	93.5±18.6	90.7±17.4	96.1±19.6	0.162
CKD-EPI SCrCys nef (mL/min/1.73m^2^)	93.5±18.1	93.3±16.7	93.7±19.5	0.902
CKD-EPI SCrCys	101.0±19.6	100.7±18.0	101.2±21.1	0.902

BMI: body mass index; BSA: body surface area, mGFR: measured glomerular filtration rate by iohexol; CKD-EPI SCr: Chronic Kidney Disease-Epidemiology Collaboration equation based on serum creatinine only, with ethnic factor; CKD-EPI SCr nef: CKD-EPI without ethnic factor; CKD-EPI SCys: CKD-EPI equation based on cystatin C only; CKD-EPI SCrCys: CKD-EPI combining creatinine and cystatin C with ethnic factor; CKD-EPISCrCys nef: CKD-EPI combining serum creatinine and cystatine C without ethnic factor; MDRD: Modification of Diet in Renal Disease study equation with ethnic factor; MDRD nef: MDRD without ethnic factor; P values for comparison between males and females.

A total of 93 healthy adult participants (mean age 45.0±15.7 years; 48 females) were included in the present study. Their BSA and body mass index (BMI) were 1.68±0.16 m^2^ and 23.5±3.40 kg/m^2^, respectively. Male participants were taller (169.0±8.1 vs 158.9±6.5 cm, p <0.001) and had lower BMI (22.6±3.1 vs 24.3±3.4; p = 0.011) than females. The mean mGFR of the participants was 92.0±17.2 mL/min/1.73m^2^. Tertiles of mGFR were defined at 84.8 and 98.3 ml/min/1.73m^2^. Mean eGFR values by different equations were: 105.6±30.1, 87.2±24.8, 109.3±24.2 and 94.3±20.9 mL/min/1.73m^2^ for MDRD, MDRD without ethnic factor (MDRD nef), CKD-EPI SCr and CKD-EPI SCr without ethnic factor (CKD-EPI SCr nef), respectively (Table 2). CKD-EPI SCys alone or combined with creatinine-based equations (with and without the ethnic factor) had the following mean values: 93.5±18.6 (CKD-EPI SCys), 93.5±18.0 (CKD-EPI SCrCys) and 101±19.6 (CKD-EPI SCrCys nef) mL/min/1.73m^2^, respectively (Table 2).

### Performance of equations

**Fig 1:**
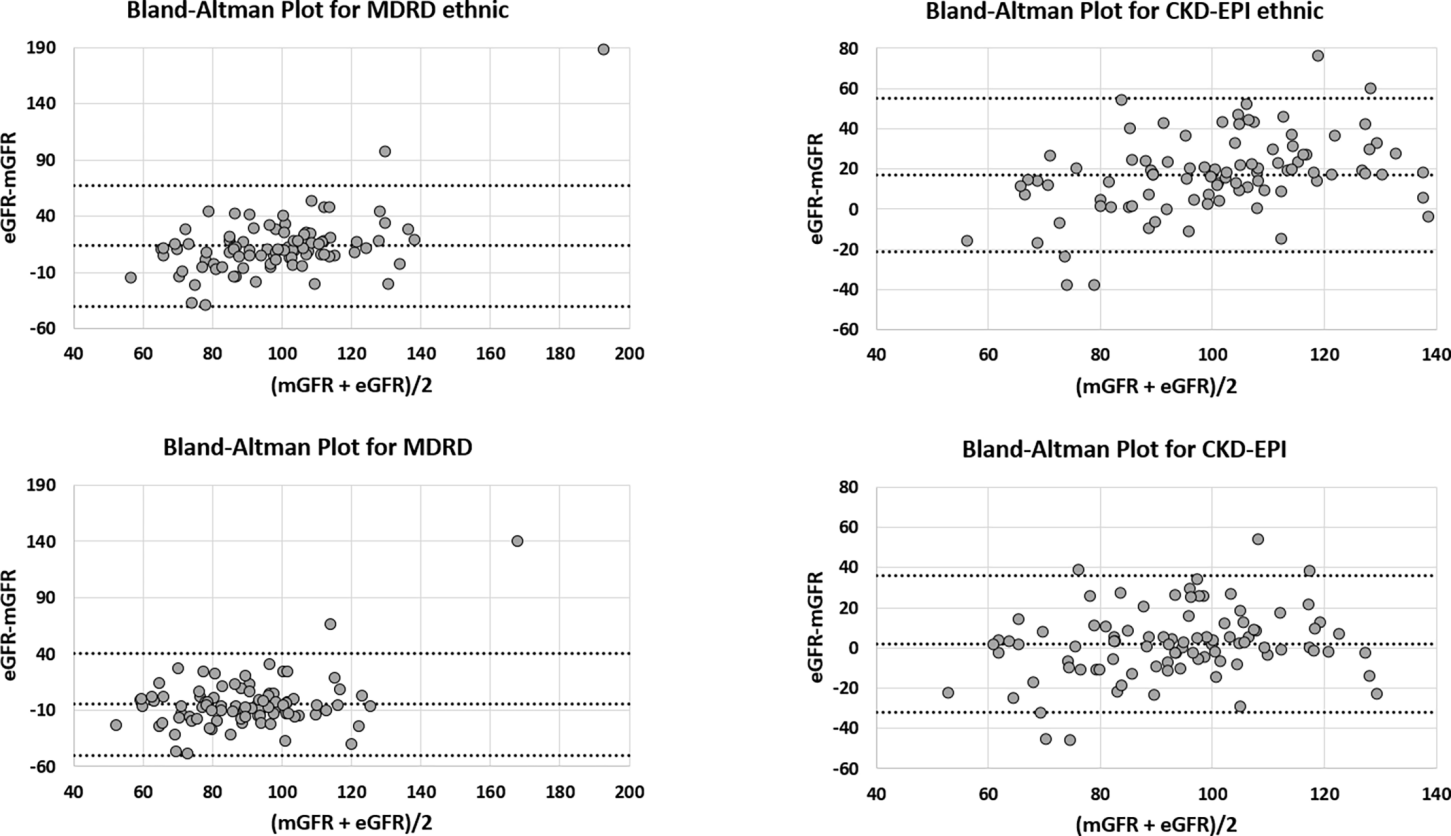
Bland and Altman analysis between mGFR and creatinine-based equations (with and without the ethnic factor). All values are expressed in mL/min/1.73m^2^. CKD-EPI SCr: Chronic Kidney Disease-Epidemiology Collaboration equation based on serum creatinine only, with ethnic factor; CKD-EPI SCr nef: CKD-EPI without ethnic factor. MDRD: Modification of Diet in Renal Disease study equation with ethnic factor; MDRD nef: MDRD without ethnic factor. The central line represents the mean difference between measured and estimated GFR, whereas the upper and lower lines represent the limits of agreement (mean difference ± 2SD).

**Fig 2:**
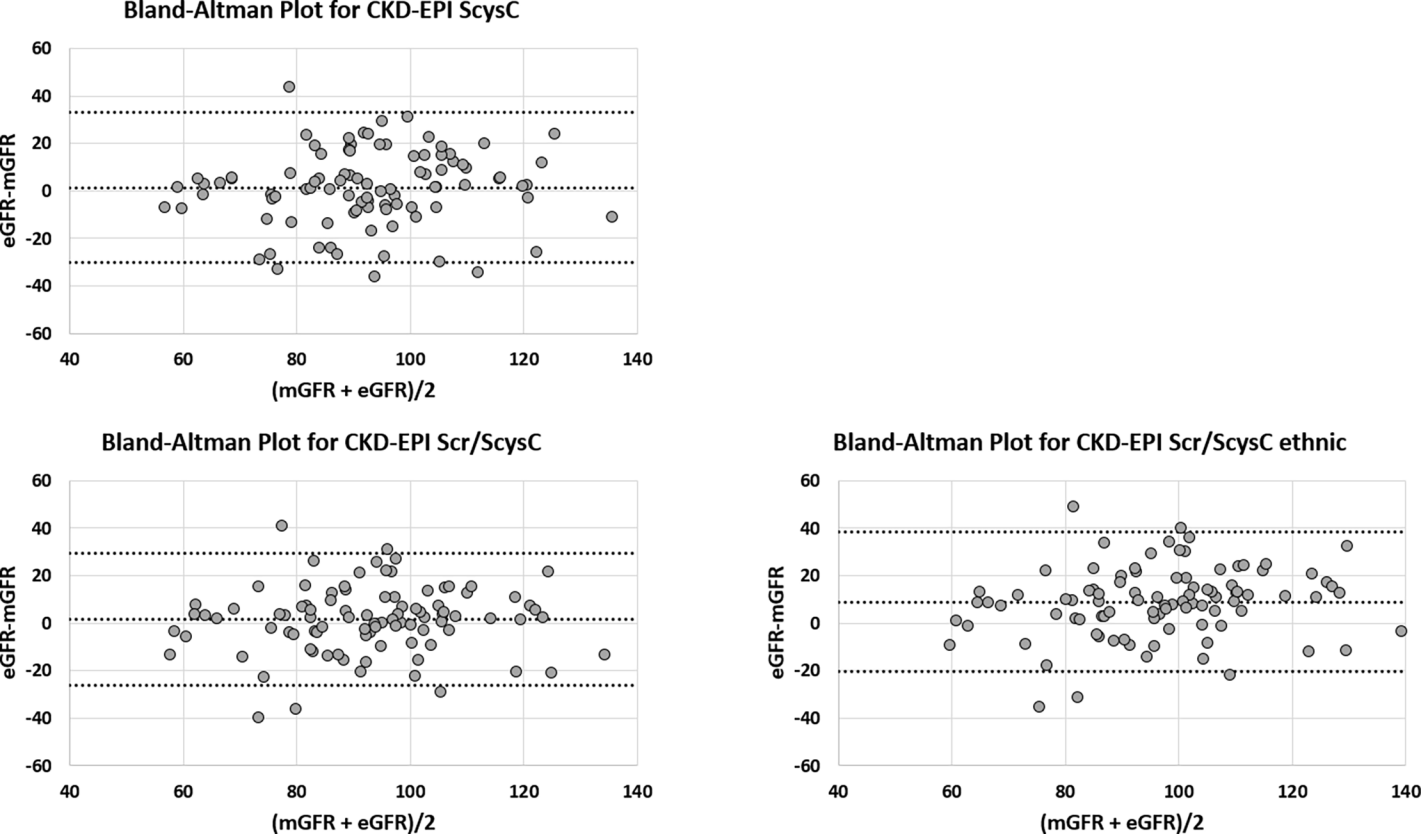
Bland and Altman analysis between mGFR and cystatin C-based and combined equations (with and without the ethnic factor). All values are expressed in mL/min/1.73m^2^. CKD-EPI SCys CKD-EPI equation based on cystatin C only; CKD-EPI SCrCys: CKD-EPI combining creatinine and cystatin C with ethnic factor. CKD-EPI SCrCys nef: CKD-EPI combining serum creatinine and cystatin C without ethnic factor. The central line represents the mean difference between measured and estimated GFR, whereas the upper and lower lines represent the limits of agreement (mean difference ± 2SD).

Table 3 summarizes the performance of the different equations whereas Figs 1 and 2 shows Bland and Altman graphics.

**Table 3:** Performance of the MDRD and CKD-EPI (with and without ethnic factors).

Equations	Lin's CCC [95%CI]	P10 [95%CI]	P30 [95%CI]	Bias [95%CI]	SD	Proportional bias [95%CI]	RMSE [95%CI]
MDRD	0.338 [0.198; 0.465]	34.4 [24.6–44.2]	79.6 [71.2–87.9]	13.6 [8.0; 19.2]	27.0	1.16 [1.10; 1.22]	30.1 [11.2; 41.1]
MDRD nef	0.414 [0.250; 0.555]	41.9 [31.7–52.2]	86.0 [78.8–94.0]	-4.9 [-9.6; -0.2]	22.9	0.96 [0.91; 1.01]	23.3 [11.0; 31.0]
CKD-EPI SCr	0.429 [0.302; 0.540]	20.4 [12.1–28.8]	73.1 [63.9–82.3]	17.2 [13.3; 21.2]	19.3	1.20 [1.15; 1.25]	25.8 [21.9; 29.2]
CKD-EPI SCr nef	0.594 [0.450; 0.707]	51.6 [41.3–62.0]	81.7 [73.7–89.7]	2.3 [-1.3; 5.8]	17.1	1.03 [0.99; 1.08]	17.1 [13.8; 20.0]
CKD-EPI SCys	0.605 [0.459; 0.718]	51.6 [41.3–62.0]	91.4 [85.6–97.2]	1.5 [-1.8; 4.7]	15.9	1.03 [0.99; 1.07]	15.9 [13.4; 18.0]
CKD-EPI SCrCys	0.607 [0.479; 0.710]	37.6 [27.6–47.7]	87.1 [80.2–94.0]	9.0 [5.9; 12.0]	14.7	1.11 [1.07; 1.15]	17.2 [14.5; 19.5]
CKD-EPI SCrCys nef	0.682 [0.557; 0.777]	57.0 [46.7–67.2]	92.5 [87.0–97.9]	1.5 [-1.4; 4.4]	14.0	1.03 [0.99; 1.06]	14.0 [11.4; 16.2]

CCC: Concordance Correlation Coefficient; CKD-EPI SCr: Chronic Kidney Disease-Epidemiology Collaboration equation based on serum creatinine only, with ethnic factor; CKD-EPI SCr nef: CKD-EPI without ethnic factor; CKD-EPI SCys: CKD-EPI equation based on cystatin C only; CKD-EPI SCrCys: CKD-EPI combining creatinine and cystatin C with ethnic factor; CKD-EPI SCrCys nef: CKD-EPI combining serum creatinine and cystatin C without ethnic factor; MDRD: Modification of Diet in Renal Disease study equation with ethnic factor; MDRD nef: MDRD without ethnic factor; P10: accuracy within 10%; P30: accuracy within 30%; RMSE: Root mean square error.

Results of Lin’s CCC showed a better result for equation without the ethnic factor compared to equation including the ethnic factor but the improvement was not statistically different. Bias of eGFRs without ethnic factor were not statistically different from equality, except for the MDRD equation which underestimated mGFR. At the opposite, a significant positive bias (eGFR overestimating mGFR) was observed for all equations with the ethnic correction.

We compared P30 and P10 in a head-to-head analysis by exact McNemar tests (Table 4).

**Table 4:** Comparison of accuracy within 30% and within 10% of estimating equations by exact McNemar tests.

		P30%	P30%	p-value	P10%	P10%	p-value
MDRD nef		86.0			41.9		
	CKD-EPI SCr nef		81.7	0.2891		51.6	0.136
	CKD-EPI SCys		91.4	0.3018		51.6	0.211
	CKD-EPI SCrCys nef		92.5	0.1094		57.0	**0.0385**
	CKD-EPI SCr		73.1	**0.0169**		20.4	**0.0091**
	CKD-EPI SCrCys		87.1	1.0000		37.6	0.6587
	MDRD		79.6	0.2101			0.4426
MDRD		79.6			34.4		
	CKD-EPI SCys		91.4	**0.0127**		51.6	**0.0226**
	CKD-EPI SCrCys nef		92.5	**0.0005**		57.0	**0.0002**
	CKD-EPI SCr		73.1	0.0703		20.4	**0.0106**
	CKD-EPI SCrCys		87.1	**0.0156**		37.6	0.7201
CKD-EPI SCr nef		81.7			51.6		
	CKD-EPI SCys		91.4	**0.0352**		51.6	1.0000
	CKD-EPI SCrCys nef		92.5	**0.0020**		57.0	0.4421
	CKD-EPI SCr		73.1	0.0574		20.4	**<0.0001**
	CKD-EPI SCrCys		87.1	0.1250		37.6	0.0533
	MDRD		79.6	0.7266		34.4	**0.0090**
CKD-EPI SCr		73.1			20.4		
	CKD-EPI SCys		91.4	**0.0002**		51.6	**<0.0001**
	CKD-EPI SCrCys nef		92.5	**<0.0001**		57.0	**<0.0001**
	CKD-EPI SCrCys		87.1	**0.0002**		37.6	**0.0004**
CKD-EPICys		91.4			51.6		
	CKD-EPI SCrCys		87.1	0.3437		37.6	0.0596
	CKD-EPI SCrCys nef		92.5	1		57	0.4049
CKD-EPI SCrCys		87.1			37.6		
	CKD-EPI SCrCys nef		92.5	**0.0039**		57	0.0625

CKD-EPI SCr: Chronic Kidney Disease-Epidemiology Collaboration equation based on serum creatinine only, with ethnic factor; CKD-EPI SCr nef: CKD-EPI without ethnic factor; CKD-EPI SCys: CKD-EPI equation based on cystatin C only; CKD-EPI SCrCys: CKD-EPI combining creatinine and cystatin C with ethnic factor. CKD-EPI SCrCys nef: CKD-EPI combining serum creatinine and cystatin C without ethnic factorMDRD: Modification of Diet in Renal Disease study equation with ethnic factor; MDRD nef: MDRD without ethnic factor. P10: accuracy within 10%; P30: accuracy within 30%; RMSE: Root mean square error.

MDRD nef had a better P30 and P10 than CKD-EPI SCr (p = 0.0169 and p = 0.0091, respectively) but a lower P10 than CKD-EPI SCrCys nef (p = 0.0385). CKD-EPI SCr nef had a better P10 than CKD-EPI SCr and MDRD (p<0.0001 and p = 0.009, respectively) but a lower P30 than CKD-EPI SCys and CKD-EPI ScrCys (p = 0.0352 p = 0.002, respectively). MDRD had a better P10 than CKD-EPI SCr (p = 0.0106) but a lower P30 than CKD-EPI SCrCys (p = 0.0156) and a lower P30 and P10 than CKD-EPI SCys and CKD-EPI ScrCys nef (p = 0.0127, p = 0.0226 and p = 0.0005 and p = 0.0002, respectively). CKD-EPI SCr had a lower P30 and P10 than CKD-EPI SCrCys (p = 0.0002 and p = 0.0004, respectively) and a lower P30 and P10 than CKD-EPI SCys and CKD-EPI ScrCys nef (p = 0.0002, p<0.0001 and p<0.0001 and p<0.0001, respectively). Finally, CKD-EPI SCrCys nef had a better P30 (p = 0.0039) than CKD-EPI SCrCys. Sub-analyses of the performances of eGFR according to gender, GFR tertiles, and age were shown in S2, S3 and S4 Files, respectively.

## Discussion

In the present study, we compared several estimating GFR equations to mGFR obtained with iohexol plasma clearance. Two main messages could be drawn from our analysis. First, bias and accuracy of equations without ethnic factors were better than corresponding equations with the ethnic factors. Second, accuracy of CKD-EPI equations integrating cystatin C was globally slightly better than equations-based on SCr sole.

We confirmed the inadequacy of African-American (AA) ethnic factors for the estimation of GFR of healthy adults in Africa and, for the first time, in Central Africa. Some authors have already criticized the AA factors both in MDRD and CKD-EPI equation. Indeed, it seems that these factors were too high, especially when applied to subjects with GFR above 60 mL/min/1.73m^2^ [28, 29, 37]. Indeed, this AA ethnic factor was determined from AA subjects, who, for the vast majority, came from the African American Study of Hypertension and Kidney Disease (AASK) and had a GFR below 60 mL/min/1.73m^2^ [38]. Moreover, differences could also occur between AA and populations living in Africa. Indeed, authors have shown that, at the same level of GFR, serum creatinine was higher in AA subjects compared to their Caucasian counterparts [39]. These differences were also observed in the African subject living in Europe, but too a much lesser extent. Comparing to a white European population, Flamant *et al* yielded that creatinine excretion and secretion rate were only slightly higher in African Europeans [28]. The ethnic factor applied to European African, including CKD patients, was thus much lower than the factor proposed originally for the AA. The relationship between SCr and GFR could however be still different in black Africans who live in a difficult socio-economic environment and not favorable to a food intake rich in creatinine-producing animal proteins. Indeed, Van Deventer *et al* have also shown that the AA ethnic factor lead to a strong overestimation of mGFR [30] in a black South-African population, including CKD patients [39]. Similar observations were also available in West-Africa (healthy people) [26, 29] (Côte d’Ivoire and Ghana). In these studies, as in ours, the performance of equations tended to be better when the AA factor was not applied, including for the CKD-EPI equation combining SCr and cystatin C. Using the AA factor also lead to very unexpected results regarding the bias of equations. Both the MDRD and CKD-EPI SCr overestimated mGFR (positive bias) when the ethnic factor was used in our healthy population. Without the ethnic factor, the bias of CKD-EPI SCr was not different and actually close to zero and the bias of MDRD became negative. These two results were totally expected in such a healthy population [17, 40].

Again, the more plausible explanations for such errors in applying the AA ethnic factor is probably the difference in muscular mass and diet (protein-rich or not) between AA and black populations living in Africa. BMI are only indirect parameters to assess muscular mass, but compared to Congolese population in the current study who had an average BMI of 23.5±3.4 kg/m2, the mean BMI observed in the AASK study cohort was much higher at 30.7 kg/m^2^ [38].

We also found that the CKD-EPI SCys equation performed better than both the MDRD and CKD-EPI SCr equation. Cystatin C is presented as a better marker of kidney function, especially because its concentration is less influenced by muscular mass or diet than SCr [41]. The best illustration is the absence of any ethnic factor in the CKD-EPI SCys equation. This observation may be of great benefit to populations living in Africa where many are from a low socioeconomic group and suffer from diseases such HIV [39]. However, in DRC, as in almost all the countries of SSA, the dosage of cystatin C is not in common use and more expensive than SCr. The slightly better performance of cystatin C suggested by our results are actually probably not enough to compensate the additional cost of this marker in daily practice.

The main conclusions of the current analysis, i.e. the better results without the ethnic factor and the slightly better performance of cystatin C-based equations remain valid in sub-analyses according to gender and GFR tertiles.

Our study has several limitations. First, it concerned only black, healthy, and Congolese subjects living in an urban environment (Kinshasa). The results need to be confirmed in CKD patients, Caucasians living in Central Africa and in rural areas. In addition, black Congolese living in developed countries, which have changed the diet and the lifestyle, was not integrated. Second, the relatively small size of our sample does not allow us to generalize our findings to the entire Congolese population. In the same view, sub-analyses results according to gender, GFR tertiles and age must be interpreted with caution because of the low sample size. Third, the results of accuracy comparisons must also be analyzed carefully because of the relatively small size of our sample and the high GFR ranges.

In conclusion, we confirmed that AA ethnic factors applied to MDRD, CKD-EPI SCr and CKD-EPI SCrCys are not suitable for a healthy population in Central Africa. Also, we suggest that cystatin C has a slightly better performance in this population. These results must be confirmed in larger cohorts, including CKD patients.

## Supporting information

S1 File Spearman correlations. CKD-EPI SCr: Chronic Kidney Disease-Epidemiology Collaboration equation based on serum creatinine only, with ethnic factor; CKD-EPI SCr nef: CKD-EPI without ethnic factor; CKD-EPI SCys: CKD-EPI equation based on cystatin C only; CKD-EPI SCrCys: CKD-EPI combining creatinine and cystatin C with ethnic factor. CKD-EPI SCrCys nef: CKD-EPI combining serum creatinine and cystatin C without ethnic factor; MDRD: Modification of Diet in Renal Disease study equation with ethnic factor; MDRD nef: MDRD without ethnic factor.
(PDF)

S2 File **Performance of the MDRD and CKD-EPI equations (with and without ethnic factors) in men (A) (n = 45) and women (B) (n = 48).**CKD-EPI SCr: Chronic Kidney Disease-Epidemiology Collaboration equation based on serum creatinine only, with ethnic factor; CKD-EPI SCr nef: CKD-EPI without ethnic factor; CKD-EPI SCys: CKD-EPI equation based on cystatin C only; CKD-EPI SCrCys: CKD-EPI combining creatinine and cystatin C with ethnic factor. CKD-EPI SCrCys nef: CKD-EPI combining serum creatinine and cystatin C without ethnic factor; MDRD: Modification of Diet in Renal Disease study equation with ethnic factor; MDRD nef: MDRD without ethnic factor; P30: accuracy within 30%; SD: Standard Deviation.
(PDF)

S3 File **Performance of the MDRD and CKD-EPI equations (with and without ethnic factors) according to tertiles of measured GFR: A: GFR<85 mL/min/1.73m**^2^ **(n = 31), B: GFR between 85 and 98 mL/min/1.73m**^2^ **(n = 31) and C: GFR > 98 mL/min/1.73m**^2^ **(n = 31).** CKD-EPI SCr: Chronic Kidney Disease-Epidemiology Collaboration equation based on serum creatinine only, with ethnic factor; CKD-EPI SCr nef: CKD-EPI without ethnic factor; CKD-EPI SCys: CKD-EPI equation based on cystatin C only; CKD-EPI SCrCys: CKD-EPI combining creatinine and cystatin C with ethnic factor. CKD-EPI SCrCys nef: CKD-EPI combining serum creatinine and cystatin C without ethnic factor; MDRD: Modification of Diet in Renal Disease study equation with ethnic factor; MDRD nef: MDRD without ethnic factor; P30: accuracy within 30%; SD: Standard Deviation.
(PDF)

S4 File **Performance of the MDRD and CKD-EPI equations (with and without ethnic factors) according to age decades: A: less than 30 years (n = 20), B: age: 30–40 years (n = 18), C: age: 40–50 years (n = 18) and D: more than 50 years (n = 37).** CKD-EPI SCr: Chronic Kidney Disease-Epidemiology Collaboration equation based on serum creatinine only, with ethnic factor; CKD-EPI SCrnef: CKD-EPI without ethnic factor; CKD-EPI SCys: CKD-EPI equation based on cystatin C only; CKD-EPI SCrCys: CKD-EPI combining creatinine and cystatin C with ethnic factor. CKD-EPI SCrCysnef: CKD-EPI combining serum creatinine and cystatine C without ethnic factor; MDRD: Modification of Diet in Renal Disease study equation with ethnic factor; MDRD nef: MDRD without ethnic factor; P30: accuracy within 30%; SD: Standard Deviation.
(PDF)

## References

[1] Levey AS, Inker LA, Coresh J. GFR estimation: from physiology to public health. *American journal of kidney diseases*: *the official journal of the National Kidney Foundation* 2014, 63(5):820–834. 24485147 10.1053/j.ajkd.2013.12.006 PMC4001724

[2] Levey AS, Atkins R, Coresh J, Cohen EP, Collins AJ, Eckardt KU et al Chronic kidney disease as a global public health problem: approaches and initiatives—a position statement from Kidney Disease Improving Global Outcomes. *Kidney international* 2007, 72(3):247–259. doi: 10.1038/sj.ki.5002343 17568785 10.1038/sj.ki.5002343

[3] Matsha TE, Yako YY, Rensburg MA, Hassan MS, Kengne AP, Erasmus RT. Chronic kidney diseases in mixed ancestry south African populations: prevalence, determinants and concordance between kidney function estimators. *BMC nephrology* 2013, 14:75 doi: 10.1186/1471-2369-14-75 23547953 10.1186/1471-2369-14-75 PMC3637389

[4] Sumaili EK, Cohen EP, Zinga CV, Krzesinski JM, Pakasa NM, Nseka NM. High prevalence of undiagnosed chronic kidney disease among at-risk population in Kinshasa, the Democratic Republic of Congo. *BMC nephrology* 2009, 10:18 doi: 10.1186/1471-2369-10-18 19622160 10.1186/1471-2369-10-18 PMC2724413

[5] Sumaili EK, Krzesinski JM, Zinga CV, Cohen EP, Delanaye P, et al Prevalence of chronic kidney disease in Kinshasa: results of a pilot study from the Democratic Republic of Congo. *Nephrology*, *dialysis*, *transplantation*: *official publication of the European Dialysis and Transplant Association—European Renal Association* 2009, 24(1):117–122. 10.1093/ndt/gfn469 18715963

[6] Stanifer JW, Jing B, Tolan S, Helmke N, Mukerjee R, Naicker S et al The epidemiology of chronic kidney disease in sub-Saharan Africa: a systematic review and meta-analysis. *The Lancet Global health* 2014, 2(3):e174–181. doi: 10.1016/S2214-109X(14)70002-6 25102850 10.1016/S2214-109X(14)70002-6

[7] Wyatt CM, Schwartz GJ, Owino Ong'or W, Abuya J, Abraham AG, Mboku C et al Estimating kidney function in HIV-infected adults in Kenya: comparison to a direct measure of glomerular filtration rate by iohexol clearance. *PloS one* 2013, 8(8):e69601 doi: 10.1371/journal.pone.0069601 23950899 10.1371/journal.pone.0069601 PMC3738577

[8] Madala ND, Thusi GP, Assounga AG, Naicker S. Characteristics of South African patients presenting with kidney disease in rural KwaZulu-Natal: a cross sectional study. *BMC nephrology* 2014, 15:61 doi: 10.1186/1471-2369-15-61 24731300 10.1186/1471-2369-15-61 PMC4003519

[9] Perico N, Remuzzi G. Need for chronic kidney disease prevention programs in disadvantaged populations. *Clinical nephrology* 2015, 83(7 Suppl 1):42–48. 25725241 10.5414/cnp83s042

[10] Katz IJ, Gerntholtz TE, van Deventer M, Schneider H, Naicker S. Is there a need for early detection programs for chronic kidney disease? *Clinical nephrology* 2010, 74 Suppl 1:S113–118. 20979975

[11] Levey AS, Eckardt KU, Tsukamoto Y, Levin A, Coresh J, Rossert J et al Definition and classification of chronic kidney disease: a position statement from Kidney Disease: Improving Global Outcomes (KDIGO). *Kidney international* 2005, 67(6):2089–2100. doi: 10.1111/j.1523-1755.2005.00365.x 15882252 10.1111/j.1523-1755.2005.00365.x

[12] Delanaye P, Ebert N, Melsom T, Gaspari F, Mariat C, Cavalier E et al Iohexol plasma clearance for measuring glomerular filtration rate in clinical practice and research: a review. Part 1: How to measure glomerular filtration rate with iohexol? *Clinical kidney journal* 2016, 9(5):682–699. doi: 10.1093/ckj/sfw070 27679715 10.1093/ckj/sfw070 PMC5036902

[13] Delanaye P, Melsom T, Ebert N, Back SE, Mariat C, Cavalier E et al Iohexol plasma clearance for measuring glomerular filtration rate in clinical practice and research: a review. Part 2: Why to measure glomerular filtration rate with iohexol? *Clinical kidney journal* 2016, 9(5):700–704. doi: 10.1093/ckj/sfw071 27679716 10.1093/ckj/sfw071 PMC5036903

[14] Cockcroft DW, Gault MH. Prediction of creatinine clearance from serum creatinine. *Nephron* 1976, 16(1):31–41. doi: 10.1159/000180580 1244564 10.1159/000180580

[15] Levey AS, Bosch JP, Lewis JB, Greene T, Rogers N, Roth D. A more accurate method to estimate glomerular filtration rate from serum creatinine: a new prediction equation. Modification of Diet in Renal Disease Study Group. *Annals of internal medicine* 1999, 130(6):461–470. 10075613 10.7326/0003-4819-130-6-199903160-00002

[16] Levey AS, Coresh J, Greene T, Stevens LA, Zhang YL, Hendriksen S et al Using standardized serum creatinine values in the modification of diet in renal disease study equation for estimating glomerular filtration rate. *Annals of internal medicine* 2006, 145(4):247–254. 16908915 10.7326/0003-4819-145-4-200608150-00004

[17] Levey AS, Stevens LA, Schmid CH, Zhang YL, Castro AF, Feldman HI et al A new equation to estimate glomerular filtration rate. *Annals of internal medicine* 2009, 150(9):604–612. 19414839 10.7326/0003-4819-150-9-200905050-00006 PMC2763564

[18] Andrassy KM. Comments on 'KDIGO 2012 Clinical Practice Guideline for the Evaluation and Management of Chronic Kidney Disease'. *Kidney international* 2013, 84(3):622–623. 10.1038/ki.2013.243 23989362

[19] Fan L, Inker LA, Rossert J, Froissart M, Rossing P, Mauer M et al Glomerular filtration rate estimation using cystatin C alone or combined with creatinine as a confirmatory test. *Nephrology*, *dialysis*, *transplantation*: *official publication of the European Dialysis and Transplant Association—European Renal Association* 2014, 29(6):1195–1203. 10.1093/ndt/gft509 PMC4471437 24449101

[20] Inker LA, Schmid CH, Tighiouart H, Eckfeldt JH, Feldman HI, Greene T et al Estimating glomerular filtration rate from serum creatinine and cystatin C. *The New England journal of medicine* 2012, 367(1):20–29. doi: 10.1056/NEJMoa1114248 22762315 10.1056/NEJMoa1114248 PMC4398023

[21] Pottel H, Delanaye P, Schaeffner E, Dubourg L, Eriksen BO, Melsom T et al Estimating glomerular filtration rate for the full age spectrum from serum creatinine and cystatin C. *Nephrology*, *dialysis*, *transplantation*: *official publication of the European Dialysis and Transplant Association—European Renal Association* 2017, 32(3):497–507. 10.1093/ndt/gfw425 PMC5837496 28089986

[22] Perrone RD, Madias NE, Levey AS. Serum creatinine as an index of renal function: new insights into old concepts. *Clinical chemistry* 1992, 38(10):1933–1953. 1394976

[23] Goldwasser P, Aboul-Magd A, Maru M. Race and creatinine excretion in chronic renal insufficiency. *American journal of kidney diseases*: *the official journal of the National Kidney Foundation* 1997, 30(1):16–22. 9214396 10.1016/s0272-6386(97)90559-x

[24] Kramer H, Palmas W, Kestenbaum B, Cushman M, Allison M, Astor B et al Chronic kidney disease prevalence estimates among racial/ethnic groups: the Multi-Ethnic Study of Atherosclerosis. *Clinical journal of the American Society of Nephrology*: *CJASN* 2008, 3(5):1391–1397. doi: 10.2215/CJN.04160907 18550650 10.2215/CJN.04160907 PMC2518793

[25] Gallagher D, Visser M, De Meersman RE, Sepulveda D, Baumgartner RN, Pierson RN et al Appendicular skeletal muscle mass: effects of age, gender, and ethnicity. *Journal of applied physiology (Bethesda*, *Md*: *1985)* 1997, 83(1):229–239. 10.1152/jappl.1997.83.1.229 9216968

[26] Eastwood JB, Kerry SM, Plange-Rhule J, Micah FB, Antwi S, Boa FG et al Assessment of GFR by four methods in adults in Ashanti, Ghana: the need for an eGFR equation for lean African populations. Nephrology, dialysis, transplantation: official publication of the European Dialysis and Transplant Association—European Renal Association 2010, 25(7):2178–2187. 10.1093/ndt/gfp765 PMC2891745 20100724

[27] Coresh J, Toto RD, Kirk KA, Whelton PK, Massry S, Jones C et al Creatinine clearance as a measure of GFR in screenees for the African-American Study of Kidney Disease and Hypertension pilot study. *American journal of kidney diseases*: *the official journal of the National Kidney Foundation* 1998, 32(1):32–42. 9669421 10.1053/ajkd.1998.v32.pm9669421

[28] Flamant M, Vidal-Petiot E, Metzger M, Haymann JP, Letavernier E, Delatour V et al Performance of GFR estimating equations in African Europeans: basis for a lower race-ethnicity factor than in African Americans. *American journal of kidney diseases*: *the official journal of the National Kidney Foundation* 2013, 62(1):182–184. 10.1053/j.ajkd.2013.03.015 23608543

[29] Sagou Yayo E, Aye M, Konan JL, Emieme A, Attoungbre ML, Gnionsahe A et al [Inadequacy of the African-American ethnic factor to estimate glomerular filtration rate in an African general population: Results from Cote d'Ivoire]. *Nephrologie & therapeutique* 2016, 12(6):454–459. 27686033 10.1016/j.nephro.2016.03.006

[30] Van Deventer HE, George JA, Paiker JE, Becker PJ, Katz IJ. Estimating glomerular filtration rate in black South Africans by use of the modification of diet in renal disease and Cockcroft-Gault equations. *Clinical chemistry* 2008, 54(7):1197–1202. doi: 10.1373/clinchem.2007.099085 18487286 10.1373/clinchem.2007.099085

[31] Sterner BF, Mansson S, Nyman U, Van Westen D, Almen T. Determining 'true' glomerular filtration rate in healthy adults using infusion of inulin and comparing it with values obtained using other clearance techniques or prediction equations. *Scandinavian Journal of Urology and Nephrology* 2008, 42:278–285. doi: 10.1080/00365590701701806 17943640 10.1080/00365590701701806

[32] Nyssen L, Delanaye P, Le Goff C, Peeters S, Cavalier E. A simple LC-MS method for the determination of iohexol and iothalamate in serum, using ioversol as an internal standard. *Clinica chimica acta; international journal of clinical chemistry* 2016, 463:96–102. doi: 10.1016/j.cca.2016.10.021 27756544 10.1016/j.cca.2016.10.021

[33] Gehan EA, George SL. Estimation of human body surface area from height and weight. *Cancer chemotherapy reports* 1970, 54(4):225–235. 5527019

[34] Ebert N, Delanaye P, Shlipak M, Jakob O, Martus P, Bartel J et al Cystatin C standardization decreases assay variation and improves assessment of glomerular filtration rate. *Clinica chimica acta; international journal of clinical chemistry* 2016, 456:115–121. doi: 10.1016/j.cca.2016.03.002 26947968 10.1016/j.cca.2016.03.002

[35] Delanaye P, Pottel H, Botev R, Inker LA, Levey AS. Con: Should we abandon the use of the MDRD equation in favour of the CKD-EPI equation? *Nephrology*, *dialysis*, *transplantation*: *official publication of the European Dialysis and Transplant Association—European Renal Association* 2013, 28(6):1396–1403; discussion 1403. 10.1093/ndt/gft006 23780677

[36] Pottel H. Critical Review of Method Comparison Studies for the Evaluation of Estimating Glomerular Filtration Rate Equations *Int J Nephrology Kidney Failure* 2015, 1(1).

[37] Delanaye P, Mariat C, Maillard N, Krzesinski JM, Cavalier E. Are the creatinine-based equations accurate to estimate glomerular filtration rate in African American populations? *Clinical journal of the American Society of Nephrology*: *CJASN* 2011, 6(4):906–912. doi: 10.2215/CJN.10931210 21441133 10.2215/CJN.10931210

[38] Lewis J, Agodoa L, Cheek D, Greene T, Middleton J, O'Connor D et al Comparison of cross-sectional renal function measurements in African Americans with hypertensive nephrosclerosis and of primary formulas to estimate glomerular filtration rate. *American journal of kidney diseases*: *the official journal of the National Kidney Foundation* 2001, 38(4):744–753. 11576877 10.1053/ajkd.2001.27691

[39] Van Deventer HE, Paiker JE, Katz IJ, George JA. A comparison of cystatin C- and creatinine-based prediction equations for the estimation of glomerular filtration rate in black South Africans. *Nephrology*, *dialysis*, *transplantation*: *official publication of the European Dialysis and Transplant Association—European Renal Association* 2011, 26(5):1553–1558. 10.1093/ndt/gfq621 PMC3108353 20961892

[40] Froissart M, Delanaye P, Seronie-Vivien S, Cristol JP. [Evaluation of renal function: an update]. *Annales de biologie clinique* 2008, 66(3):269–275. doi: 10.1684/abc.2008.0228 18558565 10.1684/abc.2008.0228

[41] Delanaye P, Cavalier E, Moranne O, Lutteri L, Krzesinski JM, Bruyere O. Creatinine-or cystatin C-based equations to estimate glomerular filtration in the general population: impact on the epidemiology of chronic kidney disease. *BMC nephrology* 2013, 14:57 doi: 10.1186/1471-2369-14-57 23496839 10.1186/1471-2369-14-57 PMC3637126

