# Supplementary material for: Performance of glomerular filtration rate estimation equations in Congolese healthy adults: The inopportunity of the ethnic correction
Source: PLoS One. 2018 Mar 2;13(3):e0193384. doi: 10.1371/journal.pone.0193384 (PMC5834186; doi:10.1371/journal.pone.0193384)
Supplement: S1 File — CKD-EPI SCr: Chronic Kidney Disease-Epidemiology Collaboration equation based on serum creatinine only, with ethnic factor; CKD-EPI SCr nef: CKD-EPI without ethnic factor; CKD-EPI SCys: CKD-EPI equation based on cystatin C only; CKD-EPI SCrCys: CKD-EPI combining creatinine and cystatin C with ethnic factor. CKD-EPI SCrCys nef: CKD-EPI combining serum creatinine and cystatin C without ethnic factor; MDRD: Modification of Diet in Renal Disease study equation with ethnic factor; MDRD nef: MDRD without ethnic factor. (PDF) [file pone.0193384.s001.pdf]

**Equations**	**Spearman correlation ρ (95% CI)**
MDRD	0.600 (0.451-0.716)
MDRD nef	0.600 (0.451-0.716)
CKD-EPI SCr	0.636 (0.490-0.740)
CKD-EPI SCr nef	0.636 (0.490-0.740)
CKD-EPI Cys	0.557 (0.399-0.684)
CKD-EPI SCrCys	0.661 (0.524-0.759)
CKD-EPI SCrCys nef	0.661 (0.525-0.760)
