# Supplementary material for: Performance of glomerular filtration rate estimation equations in Congolese healthy adults: The inopportunity of the ethnic correction
Source: PLoS One. 2018 Mar 2;13(3):e0193384. doi: 10.1371/journal.pone.0193384 (PMC5834186; doi:10.1371/journal.pone.0193384)
Supplement: S2 File — Performance of the MDRD and CKD-EPI equations (with and without ethnic factors) in men (A) (n = 45) and women (B) (n = 48).CKD-EPI SCr: Chronic Kidney Disease-Epidemiology Collaboration equation based on serum creatinine only, with ethnic factor; CKD-EPI SCr nef: CKD-EPI without ethnic factor; CKD-EPI SCys: CKD-EPI equation based on cystatin C only; CKD-EPI SCrCys: CKD-EPI combining creatinine and cystatin C with ethnic factor. CKD-EPI SCrCys nef: CKD-EPI combining serum creatinine and cystatin C without ethnic factor; MDRD: Modification of Diet in Renal Disease study equation with ethnic factor; MDRD nef: MDRD without ethnic factor; P30: accuracy within 30%; SD: Standard Deviation. (PDF) [file pone.0193384.s002.pdf]

A

Equations	Bias	SD	P30
MDRD	17	35	78
MDRD nef	-3	29	82
CKD-EPI SCr	16	21	76
CKD-EPI SCr nef	1	19	80
CKD-EPI SCys	-5	16	91
CKD-EPI SCrCys	5	15	89
CKD-EPI SCrCys nef	-2	14	93

B

Equations	Bias	SD	P30
MDRD	11	17	81
MDRD nef	-7	15	90
CKD-EPI SCr	18	17	71
CKD-EPI SCr nef	3	15	83
CKD-EPI SCys	7	14	92
CKD-EPI SCrCys	12	14	85
CKD-EPI SCrCys nef	5	13	92
