# Supplementary material for: Performance of glomerular filtration rate estimation equations in Congolese healthy adults: The inopportunity of the ethnic correction
Source: PLoS One. 2018 Mar 2;13(3):e0193384. doi: 10.1371/journal.pone.0193384 (PMC5834186; doi:10.1371/journal.pone.0193384)
Supplement: S3 File — Performance of the MDRD and CKD-EPI equations (with and without ethnic factors) according to tertiles of measured GFR: A: GFR<85 mL/min/1.73m2 (n = 31), B: GFR between 85 and 98 mL/min/1.73m2 (n = 31) and C: GFR > 98 mL/min/1.73m2 (n = 31). CKD-EPI SCr: Chronic Kidney Disease-Epidemiology Collaboration equation based on serum creatinine only, with ethnic factor; CKD-EPI SCr nef: CKD-EPI without ethnic factor; CKD-EPI SCys: CKD-EPI equation based on cystatin C only; CKD-EPI SCrCys: CKD-EPI combining creatinine and cystatin C with ethnic factor. CKD-EPI SCrCys nef: CKD-EPI combining serum creatinine and cystatin C without ethnic factor; MDRD: Modification of Diet in Renal Disease study equation with ethnic factor; MDRD nef: MDRD without ethnic factor; P30: accuracy within 30%; SD: Standard Deviation. (PDF) [file pone.0193384.s003.pdf]

A

Equations	Bias	SD	P30
MDRD	19	23	61
MDRD nef	3	19	77
CKD-EPI SCr	23	21	55
CKD-EPI SCr nef	10	18	65
CKD-EPI SCys	10	13	84
CKD-EPI SCrCys	16	14	71
CKD-EPI SCrCys nef	9	13	84

B

Equations	Bias	SD	P30
MDRD	13	38	81
MDRD nef	-6	31	87
CKD-EPI SCr	12	22	74
CKD-EPI SCr nef	-2	19	81
CKD-EPI SCys	-2	15	94
CKD-EPI SCrCys	5	14	90
CKD-EPI SCrCys nef	-3	13	94

C

Equations	Bias	SD	P30
MDRD	9	14	97
MDRD nef	-12	12	94
CKD-EPI SCr	16	13	90
CKD-EPI SCr nef	-1	12	100
CKD-EPI SCys	-4	16	97
CKD-EPI SCrCys	6	13	100

CKD-EPI SCrCys nef	-2	13	100
