# Supplementary material for: Performance of glomerular filtration rate estimation equations in Congolese healthy adults: The inopportunity of the ethnic correction
Source: PLoS One. 2018 Mar 2;13(3):e0193384. doi: 10.1371/journal.pone.0193384 (PMC5834186; doi:10.1371/journal.pone.0193384)
Supplement: S4 File — Performance of the MDRD and CKD-EPI equations (with and without ethnic factors) according to age decades: A: less than 30 years (n = 20), B: age: 30–40 years (n = 18), C: age: 40–50 years (n = 18) and D: more than 50 years (n = 37). CKD-EPI SCr: Chronic Kidney Disease-Epidemiology Collaboration equation based on serum creatinine only, with ethnic factor; CKD-EPI SCrnef: CKD-EPI without ethnic factor; CKD-EPI SCys: CKD-EPI equation based on cystatin C only; CKD-EPI SCrCys: CKD-EPI combining creatinine and cystatin C with ethnic factor. CKD-EPI SCrCysnef: CKD-EPI combining serum creatinine and cystatine C without ethnic factor; MDRD: Modification of Diet in Renal Disease study equation with ethnic factor; MDRD nef: MDRD without ethnic factor; P30: accuracy within 30%; SD: Standard Deviation. (PDF) [file pone.0193384.s004.pdf]

A

Equations	Bias	SD	P30
MDRD	20	24	75
MDRD nef	-2	22	95
CKD-EPI SCr	29	18	60
CKD-EPI SCr nef	11	17	80
CKD-EPI SCys	7	18	90
CKD-EPI SCrCys	17	15	80
CKD-EPI SCrCys nef	8	15	85

B

Equations	Bias	SD	P30
MDRD	10	15	89
MDRD nef	-10	13	94
CKD-EPI SCr	18	14	83
CKD-EPI SCr nef	2	12	89
CKD-EPI SCys	-1	16	94
CKD-EPI SCrCys	8	12	84
CKD-EPI SCrCys nef	0	12	100

C

Equations	Bias	SD	P30
MDRD	12	17	83
MDRD nef	-5	15	89
CKD-EPI SCr	20	16	78
CKD-EPI SCr nef	5	15	83
CKD-EPI SCys	8	15	83
CKD-EPI SCrCys	14	12	89
CKD-EPI SCrCys nef	6	12	94

D

Equations	Bias	SD	P30
MDRD	12	36	76
MDRD nef	-74	30	76
CKD-EPI SCr	9	21	73
CKD-EPI SCr nef	-4	18	78
CKD-EPI SCys	-3	14	95
CKD-EPI SCrCys	3	15	86
CKD-EPI SCrCys nef	-4	14	92
